# Bilateral spontaneous pneumothorax as a late complication of COVID‐19, a clinical case report

**DOI:** 10.1002/ccr3.7430

**Published:** 2023-06-02

**Authors:** Fakhri Naghavi, Fereshteh Ghiasvand, Maryam Moradi

**Affiliations:** ^1^ School of Medicine Iran University of Medical Sciences Tehran Iran; ^2^ Department of Infectious Diseases, Imam Khomeini Hospital Complex Tehran University of Medical Sciences Tehran Iran; ^3^ Eye Research Center, The Five Senses Health Institute, Rassoul Akram Hospital Iran University of Medical Sciences Tehran Iran

**Keywords:** bilateral spontaneous pneumothorax, COVID‐19, spontaneous pneumothorax

## Abstract

**Key Clinical Message:**

Bilateral spontaneous pneumothorax and sudden dyspnea can occur as late complication in patients with COVID‐19 even without any history of mechanical ventilation usage.

**Abstract:**

Bilateral spontaneous pneumothorax can occur as a late complication in patients with COVID‐19, even without any history of mechanical ventilation. Here, we present a patient with mild COVID‐19 pneumonia with a left massive pneumothorax in the third week of hospitalization and the addition of a right pneumothorax.

## Graphical abstract

Patient's early and late post‐COVID‐19 pneumothorax on chest images.

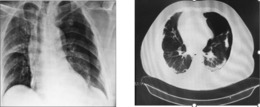

## 1 INTRODUCTION

In late 2019, a new coronavirus emerged with cases of severe pneumonia in Wuhan, China, which WHO declared its existence worldwide in February 2020 and named COVID‐19. Taxonomy international committee named this virus SARS‐COV2 (severe acute respiratory syndrome coronavirus 2) due to its similarity to the SARS pathogen in 2003. On March 11, 2020, WHO reported the new coronavirus as a pandemic.^1^

COVID‐19 symptoms are variable from mild asymptomatic to severe illness. 80% of patients have a mild‐to‐moderate form of the disease, 14% have progressive tachypnea with a decrease in oxygen saturation level and Involvement of more than 50% of the lungs, and 6% have a critical clinical condition.^2^

Bilateral peripheral ground glass opacities are the common early findings in COVID‐19 patients' imaging. Late complications such as superinfection, bacterial pneumonia, and pneumothorax can occur in several cases. Chronic obstructive pulmonary disease (COPD) and mechanical ventilation usage are the most frequent risk factors for pneumothorax in patients with COVID‐19 infection.^3^, ^4^

Pneumothorax is the accumulation of air in the space between the parietal and visceral pleura. Primary spontaneous pneumothorax occurs without any trigger, while secondary pneumothorax occurs as a complication of lung disease, lung trauma, or following mechanical ventilation. Cystic fibrosis, necrotizing pneumonia, emphysema, pulmonary fibrosis, sarcoidosis, and lung cancers are pulmonary diseases that can cause massive life‐threatening pneumothorax, which requires immediate diagnosis and drainage.^5^

Unilateral pneumothorax is an uncommon COVID‐19 complication. Bilateral spontaneous pneumothorax is an infrequent complication in patients who underwent mechanical ventilation; this side effect can occur in patients without invasive ventilation following diffuse alveolar damage caused by alveolar rupture, air leakage, and interstitial emphysema.^6^, ^7^

Patients with ARDS, having mechanical ventilation and other invasive therapies, are more likely vulnerable to pneumothorax as a late complication of COVID‐19.^8^

This case report presents a patient with severe bilateral spontaneous pneumothorax following COVID‐19 infection without any known risk factor. In this study, early pneumothorax refers to the first presentation of pneumothorax in COVID‐19 patients. Late pneumothorax refers to those symptoms that occur after several times and in the process of disease or even after proper treatment.

## 2 CASE PRESENTATION

A 41‐year‐old man without any medical history was admitted to the emergency room with sudden dyspnea and chest pain in October 2020. About 2 weeks ago, he was diagnosed with COVID‐19 infection with positive nasopharynx COVID‐PCR and, due to the appropriate clinical condition, had treated outpatient with azithromycin for 5 days (Iranian guideline for outpatient management of COVID‐19 at that time). His symptoms were dry cough, fever, and myalgia, and his oxygen saturation was 93% without any complement O_2_.

At admission, he had a dry cough with dyspnea and left hemi thorax chest pain. The patient's vital signs were as follows: (SBP: 115/72, PR: 98, RR: 22, T: 37.2, SPO_2_: 91%).

**FIGURE 1:**
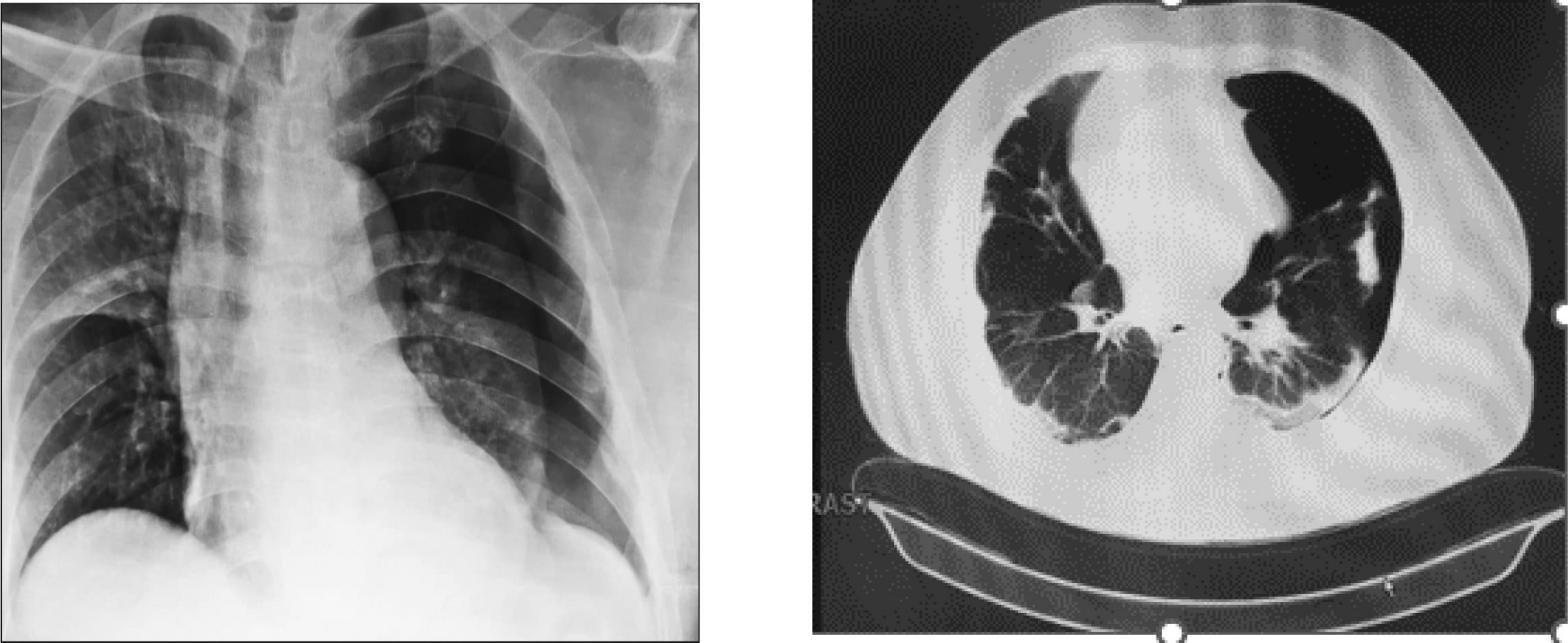
Patient's chest X‐ray and chest CT scan consisting of large left‐sided pneumothorax (early pneumothorax).

The patient had a normal ECG with left pneumothorax evidence (early pneumothorax) on his chest CT scan (Figure 1). After chest tube insertion in the 4th–5th intercostal space, he was admitted to the infectious disease ward, and further evaluations were performed. Early laboratory tests were sent, and the results are summarized in Tables 1 and 2.

**TABLE 1:** Laboratory data.

Test	Result/Unit	Reference value
WBC	**14,300 × 10** ^**9**^ **/L with 82.6% polymorphonuclear leukocytes**	4000–10,000 × 10^9^/L
HB	15.5 g/dL	13–16 g/dL
PLT	**466,000**	150,000–450,000 platelets per microliter
D‐Dimer	**1.54**	<0.5
LDH	**410**	140 units per liter (U/L) to 280 U/L
Fr	**3711.4 ng/mL**	20–250 ng/mL for adult males, 10–120 ng/mL for adult females
Troponin level	0.015 ng/mL	Between 0 and 0.4 ng/mL
CRP	**14 mg/L**	Below 3.0 mg/L
Cr	1.2 mg/dL	For adult men, 0.74–1.35 mg/dL, for adult women, 0.59–1.04 mg/dL
AST	29 IU/L	5–40 IU/L
ALT	27 IU/L	29–33 IU/L
ALK.P	**232 IU/L**	20–140 IU/L

*Note*: The values out of the normal range are bolded in the Table.

Abbreviations: ALK.P, alkaline phosphatase level; ALT, alanine transaminase level; AST, aspartate aminotransferase level; Cr, creatinine level; CRP, C‐reactive protein level; Fr, ferritin level; HB, hemoglobin level; LDH, lactate dehydrogenase level; PLT, platelet cell count; WBC, white blood cell count.

**TABLE 2:** Arterial blood gas analysis during hospitalization.

ABG	Results/Unit	Reference value
PH	7.39*	7.35–7.45
	7.29**	
	7.41***	
PCO_2_	47.1 mmHg*	35–45 mmHg
	59 mmHg**	
	37.2***	
HCO_3_	28.7 mEq/L*	22–26 mEq/L
	27.4 mEq/L**	
	23.5***	

Abbreviations: ABG, arterial blood gas; HCO_3_, bicarbonate; mEq/L, milliequivalents per liter; PCO_2_, partial pressure of carbon dioxide; PH, potential of hydrogen.

* At admission.

** On third day of hospitalization (as late pneumothorax occurs).

*** At discharge.

**FIGURE 2:**
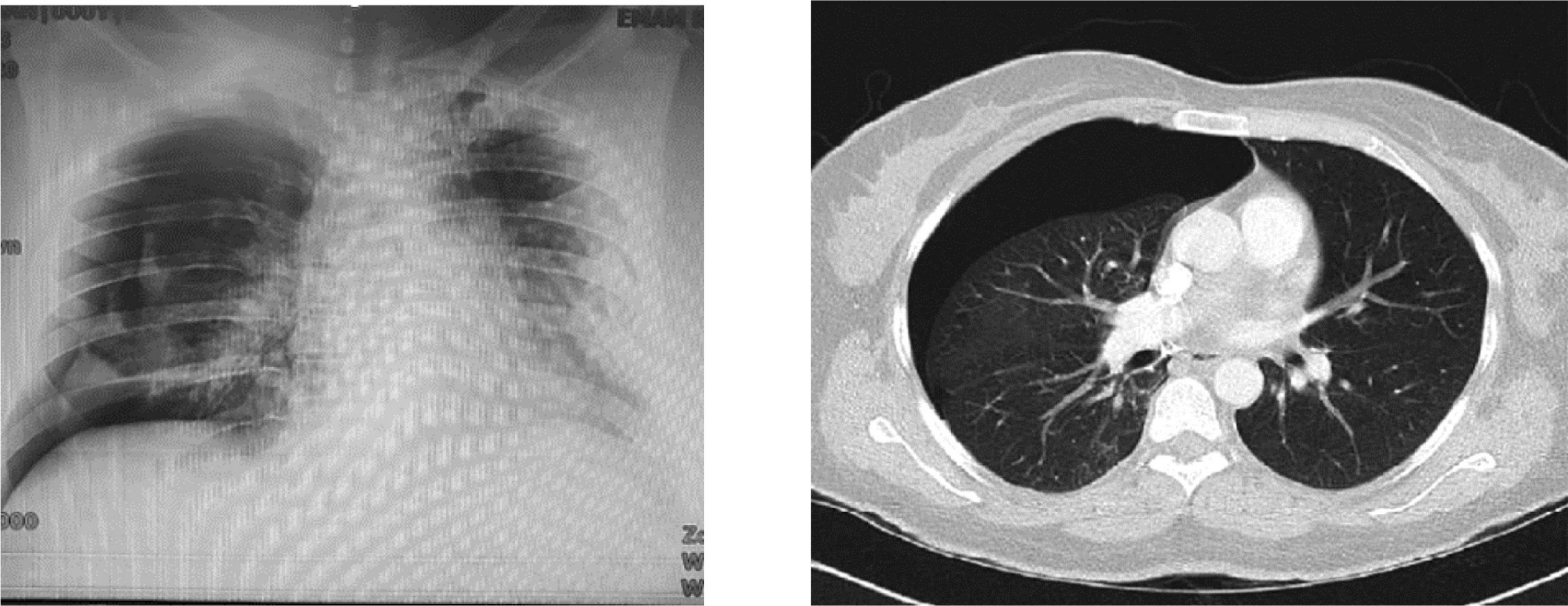
Patient's chest X‐ray and chest CT scan consisting of new right‐sided pneumothorax (late pneumothorax).

The patient was treated with 5 L/min O_2_ with a nasal cannula, 5000 units of subcutaneous heparin three times a day, 40 mg of famotidine, and 3 mg of melatonin once a day. His dyspnea and clinical condition improved on the second day of admission, but sudden right, hemi thorax chest pain, and dyspnea were started on the third day of hospitalization. Right‐sided pneumothorax (late pneumothorax) was detected after chest X‐ray evaluation. (Figure 2).

We inserted the chest tube immediately, which led to clinical improvement. Another chest X‐ray was conducted 4 days later with evidence of lung re‐expansion; therefore chest tube was removed, and the patient was discharged in stable condition. The patient does not have a recurrence of pneumothorax afterward in follow‐up sessions.

## 3 DISCUSSION

Pneumothorax is a rare, life‐threatening complication of COVID‐19 reported in several cases without any history of smoking or other pulmonary problems; therefore, it is crucial to consider this complication in patients whose clinical condition has worsened.^9^ Risk factors for primary spontaneous pneumothorax are thin, tall, male sex, age between 10 and 30, and secondary pneumothorax are smoking, infections, COPD, alpha antitrypsin deficiency, and trauma. In COVID‐19, inflammation of the airways and alveoli causes the release of cytokines and damages; this process leads to weakening of the bronchial wall, edema, vascular congestion, and micro thrombosis, which can lead to pneumothorax by rupture of the alveolar bullae.^10^

Pneumothorax, as an uncommon complication, does not occur only in patients having mechanical ventilation but also in patients in good condition at admission, so we should consider it as a late complication following COVID‐19.^11^, ^12^ In several COVID‐19 cases, pneumothorax occurs along with pneumomediastinum and subcutaneous emphysema; besides, we could not consider them as poor prognosis markers.^13^, ^14^

Bilateral spontaneous pneumothorax is an infrequent condition that occurs during COVID‐19, even in patients without invasive ventilation, presenting with sudden onset dyspnea and chest pain with a sudden decrease in oxygen saturation level. Several cases have been reported since the outbreak with the presentation of bilateral pneumothorax at admission following emphysema caused by smoking or mechanical ventilation usage due to the progressive ARDS; therefore, it is critical to detect and manage this complication immediately to prevent any further fatal consequences.^10^, ^15^, ^16^, ^17^

In our patient, the presentation started with shortness of breath and pneumothorax on the left side; despite inserting a chest tube, reducing intrathoracic pressure, and improving the clinical condition and oxygenation, the patient again had right‐sided pneumothorax on the third day of hospitalization. Compared to other cases, our case presents with one‐sided pneumothorax followed by the other side, which has not been reported.

## 4 CONCLUSION

This case report proves that bilateral spontaneous pneumothorax and sudden dyspnea can occur as late complication in patients with COVID‐19 even without any history of mechanical ventilation usage; therefore, we must keep this complication in mind to prevent its irreversible outcomes.

## AUTHOR CONTRIBUTIONS

**Fakhri Naghavi:** Conceptualization; resources. **Fereshteh Ghiasvand:** Data curation; supervision. **Maryam Moradi:** Conceptualization; supervision; writing – original draft; writing – review and editing.

## FUNDING INFORMATION

Not Applicable.

## CONFLICT OF INTEREST STATEMENT

The authors declare that they have no competing interests.

## CONSENT

The authors confirm that written consent has been obtained from the patient for submission and publication. A copy of the patient's consent for publication is available for review by the editor of the journal.

## Data Availability

Not applicable.
